# Two polymorphs of *N*,*N*′-diphenyl-2-[1-(propyl­amino)­ethyl­idene]propanedi­amide

**DOI:** 10.1107/S2056989023002141

**Published:** 2023-03-10

**Authors:** Marcus Herbig, Uwe Böhme

**Affiliations:** a Institut für Anorganische Chemie, Technische Universität Bergakademie Freiberg, Leipziger Str. 29, 09599 Freiberg, Germany; University of Kentucky, USA

**Keywords:** crystal structure, polymorph, enamine, push–pull alkene

## Abstract

Two polymorphs of the title compound, C_20_H_23_N_3_O_2_, have been isolated. Polymorph (**I**) crystallizes in the monoclinic space group *P2*_1_/*n* and polymorph (**II**) in the tetra­gonal space group *I4*_1_/*a*. The main difference between the two polymorphs on the mol­ecular level is the orientation of the *n*-propyl group. This group is anti­periplanar in (**I**) and synclinal in (**II**). The core of the mol­ecule consists of two carbamoyl units bound to an enamine unit. The most prominent features are intra­molecular N—H⋯O hydrogen bonds in both polymorphs. Both polymorphs form dimers with graph set *R*_2_^2^(12) *via* inter­molecular N—H⋯O hydrogen bonds. Adjacent dimers of (**I**) are connected *via* a weak C—H⋯O inter­action, resulting in a chain parallel to the crystallographic *a*-axis. The dimers of (**II**) are connected by weak C—H⋯π inter­actions, forming inter­molecular chains along the *c*-axis direction.

## Abstract 2

Two polymorphs of the title compound, C_20_H_23_N_3_O_2_, have been isolated. Polymorph (**I**) crystallizes in the monoclinic space group *P*2_1_/*n* and polymorph (**II**) in the tetra­gonal space group *I4*_1_/*a*. The main difference between the two polymorphs on the mol­ecular level is the orientation of the *n*-propyl group. This group is anti­periplanar in (**I**) and synclinal in (**II**).

## 1. Chemical context

*N*,*N*′-Diphenyl-2-[1-(propyl­amino)­ethyl­idene]propanedi­amide is an insertion product from an enamine and phenyl iso­cyanate. This was obtained in our work with different types of silicon–nitro­gen compounds (Herbig *et al.*, 2019*a* ▸, 2021 ▸, 2022 ▸). The Si—N bonds can be subjected to insertion of different heteroallenes such as CO_2_ and iso­cyanates (Kraushaar *et al.*, 2012 ▸, 2014 ▸, 2017 ▸; Herbig *et al.*, 2018 ▸, 2019*b* ▸). Insertion reactions into silicon-substituted enamines were investigated as a continuation of our research in this area. 
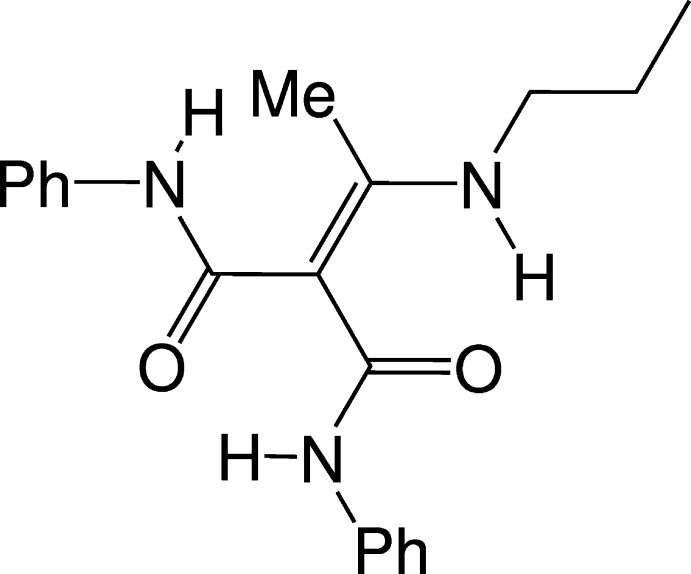


## 2. Structural commentary

**Figure 1:**
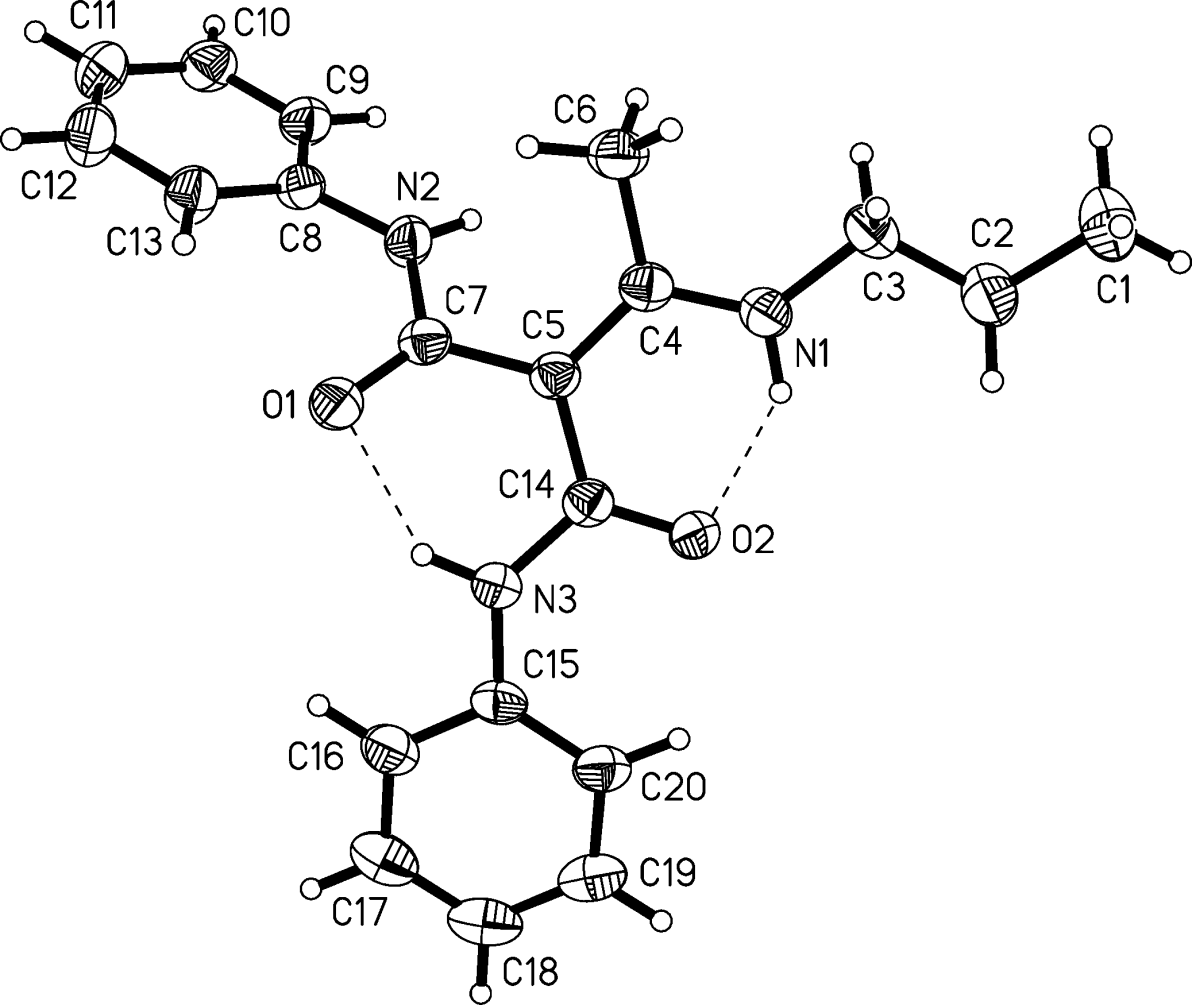
Diagram of polymorph (**I**) showing the atom-labelling scheme. Atomic displacement parameters are at the 50% probability level.

Two polymorphs of the title compound were obtained from a single reaction batch. Polymorph (**I**) crystallizes in the monoclinic space group *P*2_1_/*n*, polymorph (**II**) in the tetra­gonal space group *I4*_1_/*a*. The main difference between the polymorphs on the mol­ecular level is the orientation of the *n*-propyl group. This group is anti­periplanar in (**I**) and synclinal in (**II**), as can be seen from the values of the torsion angles C1—C2—C3—N1 (see Tables 1 ▸, 2 ▸ and Figs. 1 ▸, 2 ▸). The *n*-propyl group with C1—C2—C3 is disordered in (**II**), with site occupancies of 0.794 (7) and 0.206 (7) for parts *A* and *B*, respectively.

The double bond between C4 and C5 is slightly elongated [1.400 (2) and 1.394 (3) Å], but not as strongly as in the push–pull alkenes from cyclic ketene-*N*,*N*′-acetals, which have values of 1.45 to 1.47 Å (Ye *et al.*, 2010 ▸). The carbamoyl units are characterized by C=O double bonds and shortened C—N bonds (Tables 1 ▸ and 2 ▸), the latter having values between 1.360 (2) to 1.369 (3) Å, whereas the sum of covalent radii of C and N is 1.472 Å (Pauling, 1962 ▸).

The core of the mol­ecule consists of two carbamoyl units (N2—C7—O1 and N3—C14—O2) bound to an enamine unit (C5—C4—N1). These planar units span certain dihedral angles between each other. The dihedral angles are listed in Table 3 ▸. The dihedral angle between N1—C4—C5 and N3—C14—O2 is small in both polymorphs, with values of 10.1 (4)° in (**I**) and 8.0 (3)° in (**II**). The dihedral angles between plane N1—C4—C5 and plane N2—C7—O1 adopt larger values of 49.0 (2)° in (**I**) and 51.5 (2)° in (**II**). This means that the carbamoyl unit N2—C7—O1 is rotated further away from the enamine group than the other carbamoyl unit. Small differences between both polymorphs are found in the orientation of the phenyl groups relative to the carbamoyl units (see Table 3 ▸).

There are several intra­molecular hydrogen bonds in (**I**) and (**II**). The inter­actions N1—H1*N*⋯O2 and N3—H3*N*⋯O1 feature H⋯O distances below 2 Å (see Tables 4 ▸ and 5 ▸), which qualifies these as hydrogen bonds of moderate strength (Gilli & Gilli, 2009 ▸). Further intra­molecular inter­actions are present between C6—H6*B*⋯N2, C13—H13⋯O1, and C20—H20⋯O2 in both polymorphs.

## 3. Supra­molecular features

**Figure 3:**
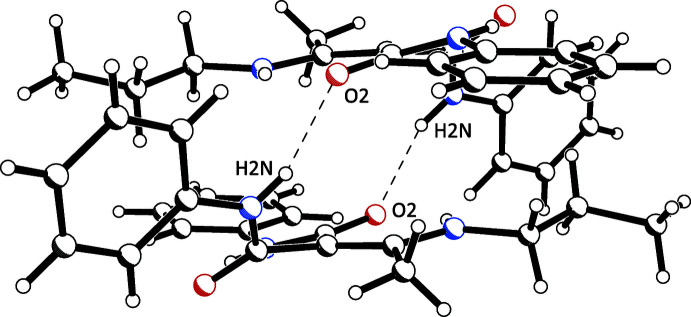
Inter­molecular N—H⋯O inter­actions leading to dimers in the crystal structure of (**I**), representative of both polymorphs.

**Figure 4:**
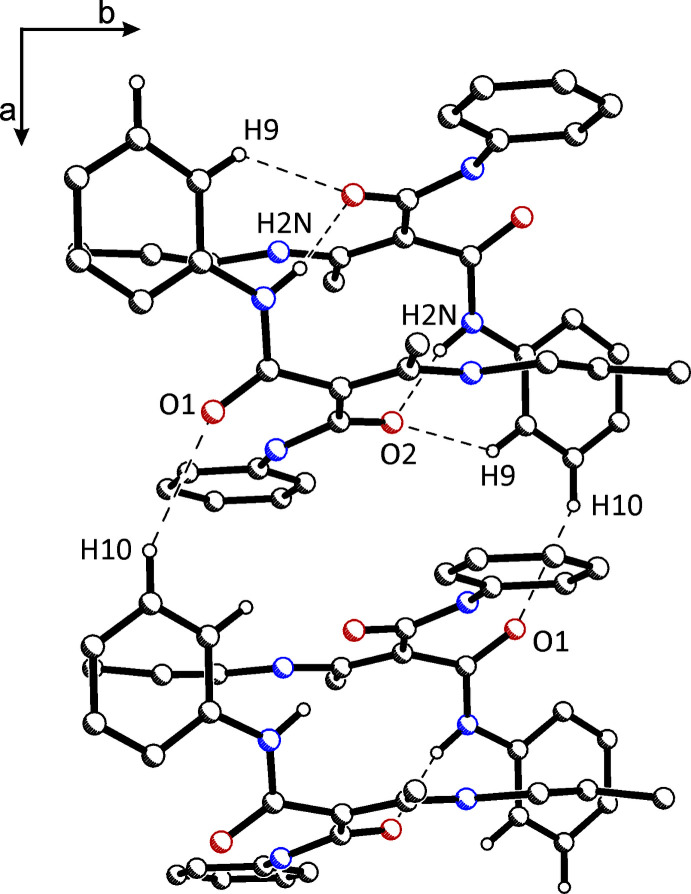
Packing diagram of polymorph (**I**) viewed along the *c* axis. Inter­molecular N—H⋯O and C—H⋯O inter­actions are shown.

**Figure 5:**
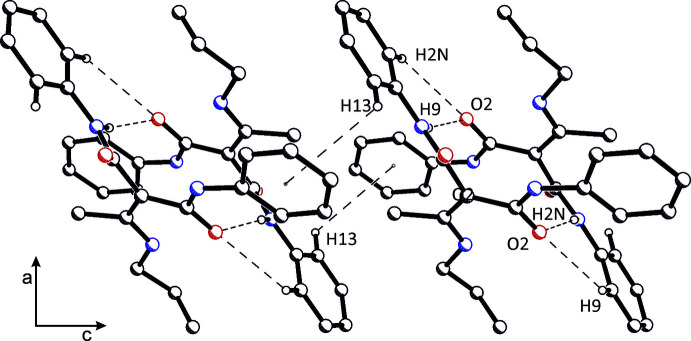
Packing diagram of polymorph (**II**) viewed along the *b* axis. Inter­molecular N—H⋯O, C—H⋯O and C—H⋯π inter­actions are shown.

The density of (**I**) is 1.243 and of (**II**) 1.235 Mg m^−3^. The mol­ecular arrangement is different in both crystals because of the crystal symmetry. The 2_1_ screw axes running parallel to the *b*-axis in the monoclinic crystal (**I**) lead to a parallel arrangement of mol­ecules in the unit cell. In contrast, in the tetra­gonal crystal of (**II**), the mol­ecules are grouped around the 4_1_ screw axes running parallel to the *c*-axis. This leads to pairs of mol­ecules that are oriented at an angle of 90° to each other. In (**I**) and (**II**), these dimers are formed by the inter­molecular N2—H2*N*⋯O2 hydrogen bonds, described by graph set 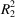 (12) (see Fig. 3 ▸). Adjacent dimers are connected by a weak C10—H10⋯O1 inter­action, resulting in a chain along the crystallographic *a*-axis direction in (**I**) (Fig. 4 ▸). In (**II**), these dimers are connected not *via* this C—H⋯O contact, but by weak C—H⋯π inter­actions, forming inter­molecular chains along the *c*-axis direction (Fig. 5 ▸). The latter are also observed in (**I**).

## 4. Database survey

Related structures are 2,2′-[benzyl­idene­methyl­enebis(carbonyl­amino)]di­benzoic acid (Taga *et al.*, 1985 ▸), bis­(*N,N*-diphen­yl)(*m*-chloro­benzyl­idene)malonyldi­amide (Kerr *et al.*, 1985 ▸; CSD refode: FACDES) and 1,1-bis­(*N*-phenyl­carbamo­yl)-2-(*p*-chloro­phen­yl)ethyl­ene (Kerr & Ashmore, 1973 ▸; CSD refode: PCMETY). Furthermore, several related push–pull alkenes from cyclic ketene-*N,N′*-acetals have been prepared and structurally characterized (Ye *et al.*, 2010 ▸). Therein, the push–pull effect reduces the double-bond order by intra­molecular charge transfer. This makes such alkenes inter­esting as substrates for second order non-linear optical materials.

## 5. Synthesis and crystallization

**Figure 6:**
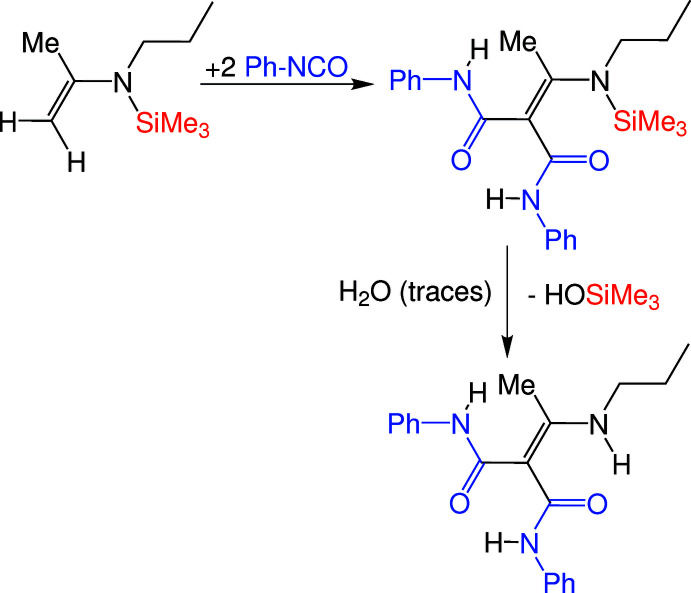
Reaction scheme for the synthesis of the title compounds.

*N*,*N*′-Diphenyl-2-[1-(propyl­amino)­ethyl­idene]propanedi­amide was obtained from the reaction of a silylated enamine (*N*-propyl-*N*-tri­methyl­silylprop-1-en-2-amine) and phenyl iso­cyanate. As shown in Fig. 6 ▸, insertion of Ph-NCO into both C—H bonds of the enamine takes place. This reaction is possible due to the lability of the β-hydrogen atoms of the enamine (Ozaki, 1972 ▸). Traces of water lead to the cleavage of the Si—N bond from the inter­mediate to yield the title compound.

To a solution of 0.46 g (3 mmol) *N*-propyl-*N*-tri­methyl­silylprop-1-en-2-amine in 10 mL of *n*-pentane was added dropwise 0.60 g (5 mmol) of phenyl­iso­cyanate at 273 K. After standing three days at room temperature, some crystals suitable for single-crystal X-ray diffraction were obtained. The polymorphs were recognised by their different crystal shapes: (**I**) forms small prisms, (**II**) forms large flat prisms. Both are colourless.

NMR spectroscopy showed that the batch product is a mixture of many components. Further purification of the product mixture was not successful.

## 6. Refinement

Crystal data, data collection and structure refinement details are summarized in Table 6 ▸. Hydrogen atoms bonded to C were positioned geometrically and allowed to ride on their parent atoms, with C—H = 0.95 Å for H(Ph), 0.99 for CH_2_, and 0.98 Å for CH_3_. *U*_iso_(H) = *xU*_eq_(C), where *x* = 1.2 for H(Ph) and CH_2_, and 1.5 for CH_3_. Hydrogen atoms on nitro­gen were localized from residual electron-density maps and were freely refined.

## Supplementary Material

Crystal structure: contains datablock(s) I, II, global. DOI: 10.1107/S2056989023002141/pk2679sup1.cif

Structure factors: contains datablock(s) I. DOI: 10.1107/S2056989023002141/pk2679Isup2.hkl

Structure factors: contains datablock(s) II. DOI: 10.1107/S2056989023002141/pk2679IIsup3.hkl

Supporting information file. DOI: 10.1107/S2056989023002141/pk2679Isup4.cml

CCDC references: 2246668, 2246667

Additional supporting information:  crystallographic information; 3D view; checkCIF report

## Figures and Tables

**Figure 2:**
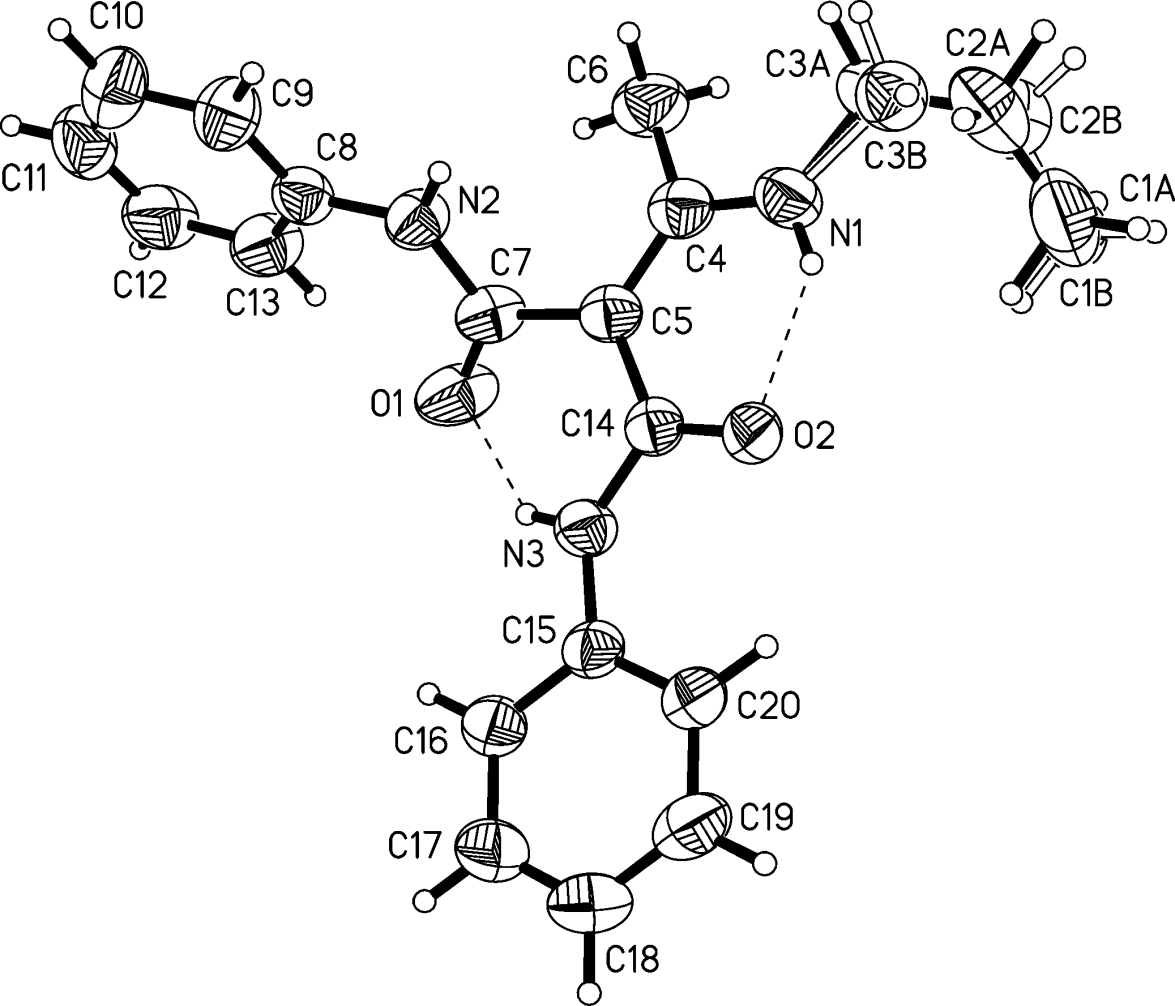
Diagram of polymorph (**II**) showing the atom-labelling scheme. Atomic displacement parameters are at the 50% probability level.

**Table 1:** Selected geometric parameters (Å, °) for (**I**)

N1—C4	1.331 (2)	C7—N2	1.364 (2)
C4—C5	1.400 (2)	C14—O2	1.254 (2)
C7—O1	1.237 (2)	C14—N3	1.360 (2)
			
C1—C2—C3—N1	−169.39 (17)		

**Table 2:** Selected geometric parameters (Å, °) for (**II**)

N1—C4	1.331 (3)	C7—N2	1.369 (3)
C4—C5	1.394 (3)	C14—O2	1.257 (2)
C7—O1	1.234 (2)	C14—N3	1.366 (2)
			
C1*A*—C2*A*—C3*A*—N1	65.0 (4)	C1*B*—C2*B*—C3*B*—N1	−46 (2)

**Table 3:** Dihedral angles (°) between selected planes in (**I**) and (**II**)

Plane 1	Plane 2	(**I**)	(**II**)
N1/C4/C5	N3/C14/O2	10.1 (4)	8.0 (3)
N1/C4/C5	N2/C7/O1	49.0 (2)	51.5 (3)
N2/C7/O1	phenyl C8–C13	26.8 (2)	23.6 (2)
N3/C14/O2	phenyl C15–C20	17.1 (3)	10.4 (2)

**Table 4:** Hydrogen-bond geometry (Å, °) for (**I**) *Cg*2 is the centroid of the C15–C20 ring.

*D*—H⋯*A*	*D*—H	H⋯*A*	*D*⋯*A*	*D*—H⋯*A*
N1—H1*N*⋯O2	0.90 (2)	1.87 (2)	2.623 (2)	139 (2)
N3—H3*N*⋯O1	0.89 (2)	1.92 (2)	2.687 (2)	142.9 (19)
C6—H6*B*⋯N2	0.98	2.45	2.943 (3)	111
C13—H13⋯O1	0.95	2.37	2.905 (2)	115
C20—H20⋯O2	0.95	2.29	2.854 (2)	117
N2—H2*N*⋯O2^i^	0.87 (2)	2.02 (2)	2.843 (2)	158 (2)
C10—H10⋯O1^ii^	0.95	2.76	3.635 (2)	154
C13—H13⋯*Cg*2^iii^	0.95	3.40	4.011 (2)	124

Symmetry codes: (i) 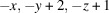 ; (ii) 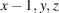 ; (iii) 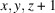 .

**Table 5:** Hydrogen-bond geometry (Å, °) for (**II**) *Cg*2 is the centroid of the C15–C20 ring.

*D*—H⋯*A*	*D*—H	H⋯*A*	*D*⋯*A*	*D*—H⋯*A*
N1—H1*N*⋯O2	0.91 (2)	1.92 (2)	2.638 (2)	135 (2)
N3—H3*N*⋯O1	0.90 (3)	1.94 (3)	2.712 (2)	144 (2)
C6—H6*B*⋯N2	0.98	2.46	2.968 (3)	112
C13—H13⋯O1	0.95	2.33	2.879 (3)	116
C20—H20⋯O2	0.95	2.30	2.887 (3)	119
N2—H2*N*⋯O2^i^	0.88 (2)	2.08 (2)	2.914 (2)	157 (2)
C9—H9⋯O2^i^	0.95	2.91	3.622 (3)	133
C13—H13⋯*Cg*2^ii^	0.95	3.37	3.960 (3)	122

Symmetry codes: (i) 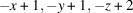 ; (ii) 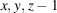 .

**Table 6:** Experimental details

	(**I**)	(**II**)
Crystal data		
Chemical formula	C_20_H_23_N_3_O_2_	C_20_H_23_N_3_O_2_
*M*_r_	337.41	337.41
Crystal system, space group	Monoclinic, *P*2_1_/*n*	Tetragonal, *I*4_1_/*a*
Temperature (K)	193	193
*a*, *b*, *c* (Å)	8.3881 (4), 24.3653 (9), 9.2614 (5)	27.7071 (11), 27.7071 (11), 9.4575 (4)
α, β, γ (°)	90, 107.724 (4), 90	90, 90, 90
*V* (Å^3^)	1802.99 (15)	7260.4 (7)
*Z*	4	16
Radiation type	Mo *K*α	Mo *K*α
μ (mm^−1^)	0.08	0.08
Crystal size (mm)	0.48 × 0.21 × 0.18	0.49 × 0.35 × 0.25

Data collection		
Diffractometer	Stoe IPDS 2	Stoe IPDS 2T
Absorption correction	Integration (*X-RED*; Stoe, 2009 ▸)	Integration (*X-RED*; Stoe, 2009 ▸)
*T*_min_, *T*_max_	0.820, 0.980	0.844, 0.980
No. of measured, independent and observed [*I* > 2σ(*I*)] reflections	18377, 3882, 2967	23893, 3884, 2635
*R*_int_	0.054	0.042
(sin θ/λ)_max_ (Å^−1^)	0.639	0.637

Refinement		
*R*[*F* ^2^ > 2σ(*F* ^2^)], *wR*(*F* ^2^), *S*	0.051, 0.129, 1.15	0.052, 0.132, 1.07
No. of reflections	3882	3884
No. of parameters	240	269
No. of restraints	0	69
H-atom treatment	H atoms treated by a mixture of independent and constrained refinement	H atoms treated by a mixture of independent and constrained refinement
Δρ_max_, Δρ_min_ (e Å^−3^)	0.14, −0.23	0.25, −0.19

Computer programs: *X-AREA* and *X-RED* (Stoe, 2009 ▸), *SHELXT* (Sheldrick, 2015*a* ▸), *SHELXL2018* (Sheldrick, 2015*b* ▸) and *ORTEP-3 for Windows* (Farrugia, 2012 ▸).

## supplementary crystallographic information

### N,N'-Diphenyl-2-[1-(propylamino)ethylidene]propanediamide (I) Crystal data

**Table:**

C_20_H_23_N_3_O_2_	*F*(000) = 720
*M**_r_* = 337.41	*D*_x_ = 1.243 Mg m^−^^3^
Monoclinic, *P*2_1_/*n*	Mo *K*α radiation, λ = 0.71073 Å
*a* = 8.3881 (4) Å	Cell parameters from 18377 reflections
*b* = 24.3653 (9) Å	θ = 2.3–27.4°
*c* = 9.2614 (5) Å	µ = 0.08 mm^−^^1^
β = 107.724 (4)°	*T* = 193 K
*V* = 1802.99 (15) Å^3^	Prism, colourless
*Z* = 4	0.48 × 0.21 × 0.18 mm

### N,N'-Diphenyl-2-[1-(propylamino)ethylidene]propanediamide (I) Data collection

**Table:**

Stoe IPDS 2 diffractometer	3882 independent reflections
Radiation source: sealed X-ray tube, 12 x 0.4 mm long-fine focus	2967 reflections with *I* > 2σ(*I*)
Detector resolution: 6.67 pixels mm^-1^	*R*_int_ = 0.054
rotation method scans	θ_max_ = 27.0°, θ_min_ = 2.5°
Absorption correction: integration (X-RED; Stoe, 2009)	*h* = −10→10
*T*_min_ = 0.820, *T*_max_ = 0.980	*k* = −30→31
18377 measured reflections	*l* = −11→11

### N,N'-Diphenyl-2-[1-(propylamino)ethylidene]propanediamide (I) Refinement

**Table:**

Refinement on *F*^2^	Primary atom site location: structure-invariant direct methods
Least-squares matrix: full	Hydrogen site location: mixed
*R*[*F*^2^ > 2σ(*F*^2^)] = 0.051	H atoms treated by a mixture of independent and constrained refinement
*wR*(*F*^2^) = 0.129	*w* = 1/[σ^2^(*F*_o_^2^) + (0.0475*P*)^2^ + 0.6249*P*] where *P* = (*F*_o_^2^ + 2*F*_c_^2^)/3
*S* = 1.15	(Δ/σ)_max_ = 0.001
3882 reflections	Δρ_max_ = 0.14 e Å^−^^3^
240 parameters	Δρ_min_ = −0.23 e Å^−^^3^
0 restraints	

### N,N'-Diphenyl-2-[1-(propylamino)ethylidene]propanediamide (I) Special details

**Table:**

Geometry. All esds (except the esd in the dihedral angle between two l.s. planes) are estimated using the full covariance matrix. The cell esds are taken into account individually in the estimation of esds in distances, angles and torsion angles; correlations between esds in cell parameters are only used when they are defined by crystal symmetry. An approximate (isotropic) treatment of cell esds is used for estimating esds involving l.s. planes.

### N,N'-Diphenyl-2-[1-(propylamino)ethylidene]propanediamide (I) Fractional atomic coordinates and isotropic or equivalent isotropic displacement parameters (Å^2^)

**Table:**

	*x*	*y*	*z*	*U*_iso_*/*U*_eq_	
C1	0.1490 (3)	1.22813 (9)	0.6450 (3)	0.0565 (6)	
H1A	0.059051	1.234402	0.690216	0.085*	
H1B	0.138400	1.254351	0.562369	0.085*	
H1C	0.257591	1.233135	0.722392	0.085*	
C2	0.1367 (3)	1.17012 (8)	0.5832 (2)	0.0457 (5)	
H2A	0.034581	1.166745	0.495147	0.055*	
H2B	0.234587	1.162517	0.547866	0.055*	
C3	0.1304 (2)	1.12830 (7)	0.7020 (2)	0.0363 (4)	
H3A	0.220902	1.135805	0.797292	0.044*	
H3B	0.021949	1.131048	0.723328	0.044*	
N1	0.15026 (19)	1.07318 (6)	0.64863 (18)	0.0342 (3)	
H1N	0.187 (3)	1.0698 (10)	0.567 (3)	0.050 (6)*	
C4	0.1460 (2)	1.02613 (7)	0.72048 (19)	0.0311 (4)	
C5	0.1863 (2)	0.97588 (7)	0.66711 (19)	0.0297 (4)	
C6	0.1047 (2)	1.03163 (8)	0.8671 (2)	0.0399 (4)	
H6A	0.175144	1.060165	0.929658	0.060*	
H6B	0.125317	0.996585	0.921533	0.060*	
H6C	−0.013406	1.041718	0.845375	0.060*	
C7	0.1491 (2)	0.92312 (7)	0.72906 (19)	0.0311 (4)	
O1	0.24257 (16)	0.88268 (6)	0.75027 (15)	0.0413 (3)	
N2	−0.00388 (19)	0.92087 (6)	0.75185 (18)	0.0336 (3)	
H2N	−0.073 (3)	0.9468 (10)	0.708 (3)	0.049 (6)*	
C8	−0.0758 (2)	0.87725 (7)	0.81029 (19)	0.0311 (4)	
C9	−0.2493 (2)	0.87627 (8)	0.7747 (2)	0.0362 (4)	
H9	−0.314313	0.903796	0.710646	0.043*	
C10	−0.3278 (2)	0.83562 (8)	0.8319 (2)	0.0416 (4)	
H10	−0.446393	0.835127	0.806474	0.050*	
C11	−0.2340 (3)	0.79569 (8)	0.9259 (2)	0.0448 (5)	
H11	−0.287648	0.767829	0.965915	0.054*	
C12	−0.0622 (3)	0.79650 (9)	0.9612 (2)	0.0452 (5)	
H12	0.002335	0.769047	1.025872	0.054*	
C13	0.0176 (2)	0.83696 (8)	0.9033 (2)	0.0390 (4)	
H13	0.136133	0.836958	0.927613	0.047*	
C14	0.25399 (19)	0.97405 (7)	0.53946 (19)	0.0299 (4)	
O2	0.25269 (15)	1.01470 (5)	0.45613 (14)	0.0342 (3)	
N3	0.3185 (2)	0.92538 (7)	0.51188 (18)	0.0359 (4)	
H3N	0.313 (3)	0.8986 (9)	0.576 (2)	0.041 (6)*	
C15	0.3593 (2)	0.90910 (8)	0.3819 (2)	0.0333 (4)	
C16	0.3682 (2)	0.85294 (9)	0.3598 (2)	0.0425 (5)	
H16	0.347921	0.828183	0.431518	0.051*	
C17	0.4061 (3)	0.83256 (10)	0.2347 (2)	0.0498 (5)	
H17	0.412200	0.794052	0.221270	0.060*	
C18	0.4352 (2)	0.86814 (10)	0.1293 (2)	0.0485 (5)	
H18	0.459997	0.854333	0.042642	0.058*	
C19	0.4277 (2)	0.92404 (10)	0.1514 (2)	0.0465 (5)	
H19	0.447291	0.948603	0.078995	0.056*	
C20	0.3919 (2)	0.94502 (8)	0.2779 (2)	0.0395 (4)	
H20	0.389836	0.983552	0.292900	0.047*	

### N,N'-Diphenyl-2-[1-(propylamino)ethylidene]propanediamide (I) Atomic displacement parameters (Å^2^)

**Table:**

	*U*^11^	*U*^22^	*U*^33^	*U*^12^	*U*^13^	*U*^23^
C1	0.0671 (15)	0.0358 (11)	0.0656 (15)	0.0032 (10)	0.0187 (12)	0.0015 (10)
C2	0.0514 (12)	0.0372 (11)	0.0476 (11)	0.0013 (9)	0.0140 (9)	−0.0014 (9)
C3	0.0352 (9)	0.0313 (9)	0.0425 (10)	0.0012 (7)	0.0122 (8)	−0.0075 (8)
N1	0.0357 (8)	0.0317 (8)	0.0375 (8)	0.0006 (6)	0.0142 (7)	−0.0033 (6)
C4	0.0251 (8)	0.0352 (9)	0.0332 (9)	−0.0010 (7)	0.0094 (7)	−0.0017 (7)
C5	0.0267 (8)	0.0323 (9)	0.0310 (8)	0.0009 (6)	0.0103 (7)	0.0006 (7)
C6	0.0433 (10)	0.0433 (11)	0.0375 (10)	0.0001 (8)	0.0186 (8)	−0.0047 (8)
C7	0.0289 (8)	0.0357 (9)	0.0298 (8)	0.0020 (7)	0.0106 (7)	0.0011 (7)
O1	0.0395 (7)	0.0422 (8)	0.0472 (8)	0.0115 (6)	0.0210 (6)	0.0118 (6)
N2	0.0292 (7)	0.0316 (8)	0.0418 (8)	0.0020 (6)	0.0137 (6)	0.0064 (7)
C8	0.0342 (9)	0.0313 (9)	0.0304 (8)	−0.0012 (7)	0.0137 (7)	−0.0011 (7)
C9	0.0334 (9)	0.0385 (10)	0.0389 (10)	0.0008 (7)	0.0143 (8)	0.0017 (8)
C10	0.0353 (10)	0.0450 (11)	0.0485 (11)	−0.0043 (8)	0.0186 (8)	−0.0006 (9)
C11	0.0471 (11)	0.0396 (11)	0.0529 (12)	−0.0077 (9)	0.0228 (9)	0.0053 (9)
C12	0.0451 (11)	0.0403 (11)	0.0505 (12)	0.0015 (8)	0.0150 (9)	0.0117 (9)
C13	0.0329 (9)	0.0402 (10)	0.0432 (10)	−0.0001 (8)	0.0106 (8)	0.0070 (8)
C14	0.0233 (7)	0.0337 (9)	0.0326 (9)	0.0006 (6)	0.0080 (6)	0.0011 (7)
O2	0.0344 (6)	0.0347 (7)	0.0373 (7)	0.0029 (5)	0.0165 (5)	0.0046 (5)
N3	0.0414 (8)	0.0329 (8)	0.0393 (8)	0.0073 (7)	0.0210 (7)	0.0036 (7)
C15	0.0255 (8)	0.0406 (10)	0.0352 (9)	0.0045 (7)	0.0115 (7)	−0.0016 (7)
C16	0.0421 (10)	0.0425 (11)	0.0459 (11)	0.0077 (8)	0.0180 (9)	−0.0017 (9)
C17	0.0481 (12)	0.0524 (13)	0.0495 (12)	0.0083 (9)	0.0158 (9)	−0.0144 (10)
C18	0.0359 (10)	0.0712 (15)	0.0390 (10)	0.0057 (10)	0.0124 (8)	−0.0138 (10)
C19	0.0372 (10)	0.0668 (14)	0.0397 (11)	0.0019 (9)	0.0181 (8)	0.0006 (10)
C20	0.0354 (10)	0.0450 (11)	0.0431 (10)	0.0026 (8)	0.0193 (8)	−0.0001 (8)

### N,N'-Diphenyl-2-[1-(propylamino)ethylidene]propanediamide (I) Geometric parameters (Å, º)

**Table:**

C1—C2	1.517 (3)	C9—C10	1.381 (3)
C1—H1A	0.9800	C9—H9	0.9500
C1—H1B	0.9800	C10—C11	1.381 (3)
C1—H1C	0.9800	C10—H10	0.9500
C2—C3	1.512 (3)	C11—C12	1.377 (3)
C2—H2A	0.9900	C11—H11	0.9500
C2—H2B	0.9900	C12—C13	1.388 (3)
C3—N1	1.458 (2)	C12—H12	0.9500
C3—H3A	0.9900	C13—H13	0.9500
C3—H3B	0.9900	C14—O2	1.254 (2)
N1—C4	1.331 (2)	C14—N3	1.360 (2)
N1—H1N	0.90 (2)	N3—C15	1.405 (2)
C4—C5	1.400 (2)	N3—H3N	0.89 (2)
C4—C6	1.506 (2)	C15—C20	1.389 (3)
C5—C14	1.460 (2)	C15—C16	1.389 (3)
C5—C7	1.479 (2)	C16—C17	1.384 (3)
C6—H6A	0.9800	C16—H16	0.9500
C6—H6B	0.9800	C17—C18	1.381 (3)
C6—H6C	0.9800	C17—H17	0.9500
C7—O1	1.237 (2)	C18—C19	1.382 (3)
C7—N2	1.364 (2)	C18—H18	0.9500
N2—C8	1.409 (2)	C19—C20	1.392 (3)
N2—H2N	0.87 (2)	C19—H19	0.9500
C8—C13	1.382 (3)	C20—H20	0.9500
C8—C9	1.391 (2)		
			
C2—C1—H1A	109.5	C9—C8—N2	117.48 (16)
C2—C1—H1B	109.5	C10—C9—C8	120.55 (18)
H1A—C1—H1B	109.5	C10—C9—H9	119.7
C2—C1—H1C	109.5	C8—C9—H9	119.7
H1A—C1—H1C	109.5	C11—C10—C9	120.02 (18)
H1B—C1—H1C	109.5	C11—C10—H10	120.0
C3—C2—C1	111.53 (18)	C9—C10—H10	120.0
C3—C2—H2A	109.3	C12—C11—C10	119.58 (18)
C1—C2—H2A	109.3	C12—C11—H11	120.2
C3—C2—H2B	109.3	C10—C11—H11	120.2
C1—C2—H2B	109.3	C11—C12—C13	120.78 (19)
H2A—C2—H2B	108.0	C11—C12—H12	119.6
N1—C3—C2	109.93 (15)	C13—C12—H12	119.6
N1—C3—H3A	109.7	C8—C13—C12	119.77 (18)
C2—C3—H3A	109.7	C8—C13—H13	120.1
N1—C3—H3B	109.7	C12—C13—H13	120.1
C2—C3—H3B	109.7	O2—C14—N3	120.18 (15)
H3A—C3—H3B	108.2	O2—C14—C5	122.81 (16)
C4—N1—C3	126.89 (16)	N3—C14—C5	117.00 (15)
C4—N1—H1N	114.0 (15)	C14—N3—C15	128.45 (16)
C3—N1—H1N	118.2 (15)	C14—N3—H3N	114.1 (14)
N1—C4—C5	122.05 (16)	C15—N3—H3N	116.0 (14)
N1—C4—C6	114.99 (16)	C20—C15—C16	119.20 (17)
C5—C4—C6	122.87 (16)	C20—C15—N3	124.55 (17)
C4—C5—C14	120.53 (15)	C16—C15—N3	116.25 (17)
C4—C5—C7	121.41 (15)	C17—C16—C15	120.9 (2)
C14—C5—C7	117.88 (15)	C17—C16—H16	119.6
C4—C6—H6A	109.5	C15—C16—H16	119.6
C4—C6—H6B	109.5	C18—C17—C16	120.1 (2)
H6A—C6—H6B	109.5	C18—C17—H17	119.9
C4—C6—H6C	109.5	C16—C17—H17	119.9
H6A—C6—H6C	109.5	C17—C18—C19	119.21 (19)
H6B—C6—H6C	109.5	C17—C18—H18	120.4
O1—C7—N2	121.60 (16)	C19—C18—H18	120.4
O1—C7—C5	123.75 (15)	C18—C19—C20	121.2 (2)
N2—C7—C5	114.50 (15)	C18—C19—H19	119.4
C7—N2—C8	128.54 (16)	C20—C19—H19	119.4
C7—N2—H2N	115.0 (15)	C15—C20—C19	119.38 (19)
C8—N2—H2N	115.2 (15)	C15—C20—H20	120.3
C13—C8—C9	119.29 (17)	C19—C20—H20	120.3
C13—C8—N2	123.20 (16)		
			
C1—C2—C3—N1	−169.39 (17)	C10—C11—C12—C13	0.0 (3)
C2—C3—N1—C4	−177.75 (17)	C9—C8—C13—C12	−0.6 (3)
C3—N1—C4—C5	−172.23 (16)	N2—C8—C13—C12	177.48 (18)
C3—N1—C4—C6	4.4 (3)	C11—C12—C13—C8	0.6 (3)
N1—C4—C5—C14	7.3 (3)	C4—C5—C14—O2	−12.5 (3)
C6—C4—C5—C14	−169.03 (16)	C7—C5—C14—O2	162.54 (15)
N1—C4—C5—C7	−167.58 (16)	C4—C5—C14—N3	168.22 (15)
C6—C4—C5—C7	16.1 (2)	C7—C5—C14—N3	−16.7 (2)
C4—C5—C7—O1	−142.19 (18)	O2—C14—N3—C15	−13.5 (3)
C14—C5—C7—O1	42.8 (2)	C5—C14—N3—C15	165.78 (17)
C4—C5—C7—N2	42.2 (2)	C14—N3—C15—C20	22.0 (3)
C14—C5—C7—N2	−132.82 (16)	C14—N3—C15—C16	−158.64 (18)
O1—C7—N2—C8	5.0 (3)	C20—C15—C16—C17	−1.1 (3)
C5—C7—N2—C8	−179.29 (16)	N3—C15—C16—C17	179.48 (18)
C7—N2—C8—C13	24.0 (3)	C15—C16—C17—C18	−0.3 (3)
C7—N2—C8—C9	−157.83 (18)	C16—C17—C18—C19	0.7 (3)
C13—C8—C9—C10	0.1 (3)	C17—C18—C19—C20	0.2 (3)
N2—C8—C9—C10	−178.08 (17)	C16—C15—C20—C19	2.0 (3)
C8—C9—C10—C11	0.4 (3)	N3—C15—C20—C19	−178.67 (17)
C9—C10—C11—C12	−0.5 (3)	C18—C19—C20—C15	−1.5 (3)

### N,N'-Diphenyl-2-[1-(propylamino)ethylidene]propanediamide (I) Hydrogen-bond geometry (Å, º)

*Cg*2 is the centroid of the C15–C20 ring.

**Table:**

*D*—H···*A*	*D*—H	H···*A*	*D*···*A*	*D*—H···*A*
N1—H1*N*···O2	0.90 (2)	1.87 (2)	2.623 (2)	139 (2)
N3—H3*N*···O1	0.89 (2)	1.92 (2)	2.687 (2)	142.9 (19)
C6—H6*B*···N2	0.98	2.45	2.943 (3)	111
C13—H13···O1	0.95	2.37	2.905 (2)	115
C20—H20···O2	0.95	2.29	2.854 (2)	117
N2—H2*N*···O2^i^	0.87 (2)	2.02 (2)	2.843 (2)	158 (2)
C10—H10···O1^ii^	0.95	2.76	3.635 (2)	154
C13—H13···*Cg*2^iii^	0.95	3.40	4.011 (2)	124

Symmetry codes: (i) −*x*, −*y*+2, −*z*+1; (ii) *x*−1, *y*, *z*; (iii) *x*, *y*, *z*+1.

### N,N'-Diphenyl-2-[1-(propylamino)ethylidene]propanediamide (II) Crystal data

**Table:**

C_20_H_23_N_3_O_2_	*D*_x_ = 1.235 Mg m^−^^3^
*M**_r_* = 337.41	Mo *K*α radiation, λ = 0.71073 Å
Tetragonal, *I*4_1_/*a*	Cell parameters from 23893 reflections
*a* = 27.7071 (11) Å	θ = 2.7–27.2°
*c* = 9.4575 (4) Å	µ = 0.08 mm^−^^1^
*V* = 7260.4 (7) Å^3^	*T* = 193 K
*Z* = 16	Prism, colourless
*F*(000) = 2880	0.49 × 0.35 × 0.25 mm

### N,N'-Diphenyl-2-[1-(propylamino)ethylidene]propanediamide (II) Data collection

**Table:**

Stoe IPDS 2T diffractometer	3884 independent reflections
Radiation source: sealed X-ray tube, 12 x 0.4 mm long-fine focus	2635 reflections with *I* > 2σ(*I*)
Detector resolution: 6.67 pixels mm^-1^	*R*_int_ = 0.042
rotation method scans	θ_max_ = 26.9°, θ_min_ = 2.7°
Absorption correction: integration (X-RED; Stoe, 2009)	*h* = −34→30
*T*_min_ = 0.844, *T*_max_ = 0.980	*k* = −34→35
23893 measured reflections	*l* = −12→11

### N,N'-Diphenyl-2-[1-(propylamino)ethylidene]propanediamide (II) Refinement

**Table:**

Refinement on *F*^2^	Primary atom site location: structure-invariant direct methods
Least-squares matrix: full	Hydrogen site location: mixed
*R*[*F*^2^ > 2σ(*F*^2^)] = 0.052	H atoms treated by a mixture of independent and constrained refinement
*wR*(*F*^2^) = 0.132	*w* = 1/[σ^2^(*F*_o_^2^) + (0.0409*P*)^2^ + 6.4556*P*] where *P* = (*F*_o_^2^ + 2*F*_c_^2^)/3
*S* = 1.07	(Δ/σ)_max_ = 0.001
3884 reflections	Δρ_max_ = 0.25 e Å^−^^3^
269 parameters	Δρ_min_ = −0.18 e Å^−^^3^
69 restraints	

### N,N'-Diphenyl-2-[1-(propylamino)ethylidene]propanediamide (II) Special details

**Table:**

Geometry. All esds (except the esd in the dihedral angle between two l.s. planes) are estimated using the full covariance matrix. The cell esds are taken into account individually in the estimation of esds in distances, angles and torsion angles; correlations between esds in cell parameters are only used when they are defined by crystal symmetry. An approximate (isotropic) treatment of cell esds is used for estimating esds involving l.s. planes.

### N,N'-Diphenyl-2-[1-(propylamino)ethylidene]propanediamide (II) Fractional atomic coordinates and isotropic or equivalent isotropic displacement parameters (Å^2^)

**Table:**

	*x*	*y*	*z*	*U*_iso_*/*U*_eq_	Occ. (<1)
C1A	0.33034 (19)	0.4509 (2)	1.0073 (5)	0.1009 (15)	0.794 (7)
H1A	0.345612	0.480495	1.042488	0.151*	0.794 (7)
H1B	0.315992	0.433244	1.086538	0.151*	0.794 (7)
H1C	0.305092	0.459356	0.939020	0.151*	0.794 (7)
C2A	0.36613 (16)	0.42112 (13)	0.9393 (4)	0.0792 (12)	0.794 (7)
H2A	0.349729	0.392523	0.898911	0.095*	0.794 (7)
H2B	0.388915	0.409434	1.012267	0.095*	0.794 (7)
C3A	0.39475 (11)	0.44513 (12)	0.8236 (4)	0.0557 (9)	0.794 (7)
H3A	0.416173	0.421327	0.777213	0.067*	0.794 (7)
H3B	0.372703	0.458651	0.751383	0.067*	0.794 (7)
C1B	0.3142 (5)	0.4689 (5)	0.9652 (16)	0.064 (4)	0.206 (7)
H1D	0.335316	0.489802	1.021394	0.095*	0.206 (7)
H1E	0.294887	0.448625	1.028587	0.095*	0.206 (7)
H1F	0.292641	0.488873	0.907398	0.095*	0.206 (7)
C2B	0.3429 (5)	0.4388 (5)	0.8749 (18)	0.082 (4)	0.206 (7)
H2C	0.334739	0.448684	0.777208	0.098*	0.206 (7)
H2D	0.329972	0.405792	0.886790	0.098*	0.206 (7)
C3B	0.3960 (4)	0.4337 (4)	0.8773 (17)	0.060 (4)	0.206 (7)
H3C	0.406400	0.416664	0.790496	0.072*	0.206 (7)
H3D	0.405204	0.413461	0.959305	0.072*	0.206 (7)
N1	0.42335 (7)	0.48356 (7)	0.8871 (2)	0.0554 (5)	
H1N	0.4203 (8)	0.4899 (8)	0.981 (3)	0.062 (7)*	
C4	0.45371 (7)	0.51141 (7)	0.8148 (2)	0.0489 (5)	
C5	0.47728 (7)	0.55038 (7)	0.8774 (2)	0.0441 (4)	
C6	0.45777 (9)	0.50023 (10)	0.6592 (2)	0.0673 (6)	
H6A	0.474937	0.469611	0.646512	0.101*	
H6B	0.475621	0.526138	0.611807	0.101*	
H6C	0.425402	0.497686	0.618089	0.101*	
C7	0.51595 (8)	0.57654 (7)	0.8002 (2)	0.0503 (5)	
O1	0.51926 (7)	0.62093 (5)	0.79818 (17)	0.0724 (5)	
N2	0.54997 (6)	0.54761 (6)	0.73837 (19)	0.0496 (4)	
H2N	0.5496 (8)	0.5170 (8)	0.764 (2)	0.055 (6)*	
C8	0.59020 (8)	0.56177 (7)	0.6563 (2)	0.0486 (5)	
C9	0.62718 (8)	0.52880 (9)	0.6397 (2)	0.0607 (6)	
H9	0.625036	0.497964	0.683170	0.073*	
C10	0.66752 (9)	0.54064 (11)	0.5594 (3)	0.0747 (7)	
H10	0.692931	0.517901	0.548843	0.090*	
C11	0.67088 (10)	0.58508 (11)	0.4952 (3)	0.0752 (7)	
H11	0.698696	0.593338	0.441738	0.090*	
C12	0.63374 (11)	0.61724 (9)	0.5093 (3)	0.0758 (8)	
H12	0.635694	0.647678	0.463431	0.091*	
C13	0.59336 (10)	0.60625 (8)	0.5892 (3)	0.0660 (6)	
H13	0.567914	0.629032	0.598067	0.079*	
C14	0.46565 (7)	0.56580 (6)	1.0214 (2)	0.0409 (4)	
O2	0.43901 (5)	0.54185 (4)	1.10290 (14)	0.0463 (3)	
N3	0.48566 (6)	0.60834 (6)	1.06502 (18)	0.0480 (4)	
H3N	0.5014 (9)	0.6240 (9)	0.996 (3)	0.069 (7)*	
C15	0.48675 (7)	0.62908 (7)	1.2011 (2)	0.0441 (4)	
C16	0.51880 (8)	0.66737 (7)	1.2202 (2)	0.0539 (5)	
H16	0.538506	0.677647	1.143708	0.065*	
C17	0.52215 (9)	0.69048 (8)	1.3491 (3)	0.0652 (6)	
H17	0.543793	0.716791	1.360352	0.078*	
C18	0.49444 (10)	0.67569 (9)	1.4611 (3)	0.0726 (7)	
H18	0.496925	0.691430	1.550003	0.087*	
C19	0.46305 (10)	0.63785 (9)	1.4430 (3)	0.0714 (7)	
H19	0.443956	0.627485	1.520741	0.086*	
C20	0.45852 (8)	0.61441 (8)	1.3144 (2)	0.0556 (5)	
H20	0.436309	0.588537	1.303791	0.067*	

### N,N'-Diphenyl-2-[1-(propylamino)ethylidene]propanediamide (II) Atomic displacement parameters (Å^2^)

**Table:**

	*U*^11^	*U*^22^	*U*^33^	*U*^12^	*U*^13^	*U*^23^
C1A	0.092 (3)	0.118 (3)	0.093 (3)	−0.055 (2)	0.009 (2)	−0.002 (2)
C2A	0.0903 (19)	0.0684 (16)	0.0790 (18)	−0.0305 (14)	−0.0128 (15)	0.0055 (13)
C3A	0.0578 (14)	0.0513 (14)	0.0580 (15)	−0.0049 (11)	−0.0094 (12)	−0.0071 (12)
C1B	0.060 (4)	0.060 (5)	0.071 (5)	−0.001 (3)	0.005 (3)	0.003 (3)
C2B	0.081 (5)	0.082 (5)	0.084 (5)	−0.001 (2)	0.000 (2)	−0.004 (2)
C3B	0.060 (4)	0.061 (4)	0.059 (4)	−0.001 (2)	−0.002 (2)	−0.002 (2)
N1	0.0518 (10)	0.0524 (10)	0.0620 (12)	−0.0028 (8)	−0.0085 (9)	−0.0123 (9)
C4	0.0460 (11)	0.0499 (11)	0.0508 (11)	0.0097 (9)	−0.0066 (9)	−0.0037 (9)
C5	0.0471 (11)	0.0411 (10)	0.0440 (10)	0.0058 (8)	−0.0026 (9)	0.0001 (8)
C6	0.0674 (15)	0.0805 (16)	0.0539 (13)	0.0079 (12)	−0.0086 (11)	−0.0185 (12)
C7	0.0650 (13)	0.0431 (11)	0.0428 (10)	0.0069 (9)	0.0047 (10)	0.0027 (9)
O1	0.1109 (14)	0.0413 (8)	0.0649 (10)	0.0050 (8)	0.0316 (10)	0.0050 (7)
N2	0.0557 (10)	0.0402 (9)	0.0528 (10)	0.0032 (7)	0.0092 (8)	0.0039 (8)
C8	0.0553 (12)	0.0474 (11)	0.0432 (10)	−0.0078 (9)	0.0026 (9)	−0.0018 (9)
C9	0.0566 (13)	0.0656 (14)	0.0598 (13)	0.0030 (11)	0.0044 (11)	0.0078 (11)
C10	0.0574 (14)	0.094 (2)	0.0726 (16)	0.0023 (13)	0.0121 (13)	0.0023 (15)
C11	0.0698 (17)	0.0887 (19)	0.0671 (15)	−0.0283 (15)	0.0151 (13)	−0.0060 (14)
C12	0.100 (2)	0.0582 (14)	0.0691 (16)	−0.0249 (14)	0.0257 (15)	−0.0036 (12)
C13	0.0837 (17)	0.0497 (12)	0.0646 (14)	−0.0042 (11)	0.0186 (13)	0.0022 (11)
C14	0.0398 (10)	0.0369 (9)	0.0459 (10)	0.0048 (7)	−0.0006 (8)	0.0017 (8)
O2	0.0458 (7)	0.0411 (7)	0.0521 (8)	−0.0017 (6)	0.0064 (6)	−0.0013 (6)
N3	0.0581 (10)	0.0397 (9)	0.0462 (9)	−0.0063 (7)	0.0079 (8)	−0.0011 (7)
C15	0.0453 (10)	0.0369 (10)	0.0501 (11)	0.0060 (8)	0.0033 (9)	−0.0027 (8)
C16	0.0558 (12)	0.0415 (11)	0.0645 (13)	−0.0025 (9)	0.0075 (10)	−0.0076 (10)
C17	0.0664 (15)	0.0504 (12)	0.0789 (16)	−0.0022 (11)	0.0022 (13)	−0.0192 (12)
C18	0.0843 (18)	0.0642 (15)	0.0694 (16)	0.0026 (13)	0.0052 (14)	−0.0263 (13)
C19	0.0795 (17)	0.0749 (16)	0.0598 (14)	−0.0014 (13)	0.0221 (13)	−0.0151 (13)
C20	0.0565 (12)	0.0522 (12)	0.0580 (13)	−0.0043 (9)	0.0133 (10)	−0.0099 (10)

### N,N'-Diphenyl-2-[1-(propylamino)ethylidene]propanediamide (II) Geometric parameters (Å, º)

**Table:**

C1A—C2A	1.442 (6)	C7—N2	1.369 (3)
C1A—H1A	0.9800	N2—C8	1.414 (3)
C1A—H1B	0.9800	N2—H2N	0.88 (2)
C1A—H1C	0.9800	C8—C9	1.382 (3)
C2A—C3A	1.506 (5)	C8—C13	1.389 (3)
C2A—H2A	0.9900	C9—C10	1.390 (3)
C2A—H2B	0.9900	C9—H9	0.9500
C3A—N1	1.457 (3)	C10—C11	1.376 (4)
C3A—H3A	0.9900	C10—H10	0.9500
C3A—H3B	0.9900	C11—C12	1.368 (4)
C1B—C2B	1.435 (14)	C11—H11	0.9500
C1B—H1D	0.9800	C12—C13	1.384 (3)
C1B—H1E	0.9800	C12—H12	0.9500
C1B—H1F	0.9800	C13—H13	0.9500
C2B—C3B	1.477 (14)	C14—O2	1.257 (2)
C2B—H2C	0.9900	C14—N3	1.366 (2)
C2B—H2D	0.9900	N3—C15	1.410 (3)
C3B—N1	1.580 (12)	N3—H3N	0.90 (3)
C3B—H3C	0.9900	C15—C20	1.387 (3)
C3B—H3D	0.9900	C15—C16	1.395 (3)
N1—C4	1.331 (3)	C16—C17	1.380 (3)
N1—H1N	0.91 (2)	C16—H16	0.9500
C4—C5	1.394 (3)	C17—C18	1.370 (4)
C4—C6	1.508 (3)	C17—H17	0.9500
C5—C14	1.463 (3)	C18—C19	1.373 (4)
C5—C7	1.485 (3)	C18—H18	0.9500
C6—H6A	0.9800	C19—C20	1.385 (3)
C6—H6B	0.9800	C19—H19	0.9500
C6—H6C	0.9800	C20—H20	0.9500
C7—O1	1.234 (2)		
			
C2A—C1A—H1A	109.5	H6A—C6—H6C	109.5
C2A—C1A—H1B	109.5	H6B—C6—H6C	109.5
H1A—C1A—H1B	109.5	O1—C7—N2	121.7 (2)
C2A—C1A—H1C	109.5	O1—C7—C5	123.24 (19)
H1A—C1A—H1C	109.5	N2—C7—C5	114.91 (17)
H1B—C1A—H1C	109.5	C7—N2—C8	127.97 (18)
C1A—C2A—C3A	115.7 (3)	C7—N2—H2N	115.9 (14)
C1A—C2A—H2A	108.4	C8—N2—H2N	115.3 (14)
C3A—C2A—H2A	108.4	C9—C8—C13	119.2 (2)
C1A—C2A—H2B	108.4	C9—C8—N2	117.63 (18)
C3A—C2A—H2B	108.4	C13—C8—N2	123.1 (2)
H2A—C2A—H2B	107.4	C8—C9—C10	120.2 (2)
N1—C3A—C2A	108.1 (3)	C8—C9—H9	119.9
N1—C3A—H3A	110.1	C10—C9—H9	119.9
C2A—C3A—H3A	110.1	C11—C10—C9	120.4 (3)
N1—C3A—H3B	110.1	C11—C10—H10	119.8
C2A—C3A—H3B	110.1	C9—C10—H10	119.8
H3A—C3A—H3B	108.4	C12—C11—C10	119.3 (2)
C2B—C1B—H1D	109.5	C12—C11—H11	120.4
C2B—C1B—H1E	109.5	C10—C11—H11	120.4
H1D—C1B—H1E	109.5	C11—C12—C13	121.2 (2)
C2B—C1B—H1F	109.5	C11—C12—H12	119.4
H1D—C1B—H1F	109.5	C13—C12—H12	119.4
H1E—C1B—H1F	109.5	C12—C13—C8	119.7 (2)
C1B—C2B—C3B	126.9 (13)	C12—C13—H13	120.1
C1B—C2B—H2C	105.6	C8—C13—H13	120.1
C3B—C2B—H2C	105.6	O2—C14—N3	120.55 (18)
C1B—C2B—H2D	105.6	O2—C14—C5	123.10 (17)
C3B—C2B—H2D	105.6	N3—C14—C5	116.35 (17)
H2C—C2B—H2D	106.1	C14—N3—C15	129.51 (17)
C2B—C3B—N1	113.2 (10)	C14—N3—H3N	113.2 (16)
C2B—C3B—H3C	108.9	C15—N3—H3N	117.2 (16)
N1—C3B—H3C	108.9	C20—C15—C16	118.78 (19)
C2B—C3B—H3D	108.9	C20—C15—N3	125.00 (18)
N1—C3B—H3D	108.9	C16—C15—N3	116.23 (18)
H3C—C3B—H3D	107.8	C17—C16—C15	120.7 (2)
C4—N1—C3A	123.8 (2)	C17—C16—H16	119.7
C4—N1—C3B	141.3 (6)	C15—C16—H16	119.7
C4—N1—H1N	116.8 (15)	C18—C17—C16	120.4 (2)
C3A—N1—H1N	119.5 (15)	C18—C17—H17	119.8
C3B—N1—H1N	100.4 (16)	C16—C17—H17	119.8
N1—C4—C5	121.78 (19)	C17—C18—C19	119.2 (2)
N1—C4—C6	115.5 (2)	C17—C18—H18	120.4
C5—C4—C6	122.6 (2)	C19—C18—H18	120.4
C4—C5—C14	121.24 (18)	C18—C19—C20	121.6 (2)
C4—C5—C7	120.47 (18)	C18—C19—H19	119.2
C14—C5—C7	118.27 (17)	C20—C19—H19	119.2
C4—C6—H6A	109.5	C19—C20—C15	119.3 (2)
C4—C6—H6B	109.5	C19—C20—H20	120.3
H6A—C6—H6B	109.5	C15—C20—H20	120.3
C4—C6—H6C	109.5		
			
C1A—C2A—C3A—N1	65.0 (4)	C8—C9—C10—C11	−0.4 (4)
C1B—C2B—C3B—N1	−46 (2)	C9—C10—C11—C12	−1.0 (4)
C2A—C3A—N1—C4	178.5 (3)	C10—C11—C12—C13	1.3 (4)
C2B—C3B—N1—C4	−116.6 (12)	C11—C12—C13—C8	−0.2 (4)
C3A—N1—C4—C5	174.5 (2)	C9—C8—C13—C12	−1.3 (4)
C3B—N1—C4—C5	−166.6 (8)	N2—C8—C13—C12	−179.3 (2)
C3A—N1—C4—C6	−1.1 (3)	C4—C5—C14—O2	9.5 (3)
C3B—N1—C4—C6	17.9 (9)	C7—C5—C14—O2	−168.94 (17)
N1—C4—C5—C14	−7.0 (3)	C4—C5—C14—N3	−170.85 (17)
C6—C4—C5—C14	168.25 (18)	C7—C5—C14—N3	10.7 (2)
N1—C4—C5—C7	171.42 (18)	O2—C14—N3—C15	9.3 (3)
C6—C4—C5—C7	−13.4 (3)	C5—C14—N3—C15	−170.35 (18)
C4—C5—C7—O1	136.4 (2)	C14—N3—C15—C20	−14.1 (3)
C14—C5—C7—O1	−45.1 (3)	C14—N3—C15—C16	166.21 (19)
C4—C5—C7—N2	−47.7 (3)	C20—C15—C16—C17	−0.4 (3)
C14—C5—C7—N2	130.73 (19)	N3—C15—C16—C17	179.2 (2)
O1—C7—N2—C8	−5.4 (3)	C15—C16—C17—C18	0.9 (4)
C5—C7—N2—C8	178.67 (19)	C16—C17—C18—C19	−0.5 (4)
C7—N2—C8—C9	161.2 (2)	C17—C18—C19—C20	−0.3 (4)
C7—N2—C8—C13	−20.8 (3)	C18—C19—C20—C15	0.7 (4)
C13—C8—C9—C10	1.6 (3)	C16—C15—C20—C19	−0.3 (3)
N2—C8—C9—C10	179.6 (2)	N3—C15—C20—C19	180.0 (2)

### N,N'-Diphenyl-2-[1-(propylamino)ethylidene]propanediamide (II) Hydrogen-bond geometry (Å, º)

*Cg*2 is the centroid of the C15–C20 ring.

**Table:**

*D*—H···*A*	*D*—H	H···*A*	*D*···*A*	*D*—H···*A*
N1—H1*N*···O2	0.91 (2)	1.92 (2)	2.638 (2)	135 (2)
N3—H3*N*···O1	0.90 (3)	1.94 (3)	2.712 (2)	144 (2)
C6—H6*B*···N2	0.98	2.46	2.968 (3)	112
C13—H13···O1	0.95	2.33	2.879 (3)	116
C20—H20···O2	0.95	2.30	2.887 (3)	119
N2—H2*N*···O2^i^	0.88 (2)	2.08 (2)	2.914 (2)	157 (2)
C9—H9···O2^i^	0.95	2.91	3.622 (3)	133
C13—H13···*Cg*2^ii^	0.95	3.37	3.960 (3)	122

Symmetry codes: (i) −*x*+1, −*y*+1, −*z*+2; (ii) *x*, *y*, *z*−1.
